# Trends in clinical characteristics and outcomes of all critically ill COVID-19 adult patients hospitalized in France between March 2020 and June 2021: a national database study

**DOI:** 10.1186/s13613-022-01097-3

**Published:** 2023-01-12

**Authors:** Diane Naouri, Albert Vuagnat, Gaëtan Beduneau, Martin Dres, Tai Pham, Alain Mercat, Alain Combes, Alexandre Demoule, Antoine Kimmoun, Matthieu Schmidt, Matthieu Jamme

**Affiliations:** 1 Department for Research, Studies, Assessment and Statistics (DREES), French Ministry of Health, 10 Place Des 5 Martyrs du Lycée Buffon, 75014 Paris, France; 2 UNIROUEN, EA 3830, Medical Intensive Care Unit, Rouen University Hospital, Normandie University, 76000 Rouen, France; 3 Service de Pneumologie et Réanimation Médicale, Hôpital Pitié Salpétrière, Assistance Publique Hôpitaux de Paris, Paris, France; 4 Service de Médecine Intensive-Réanimation, Hôpital du Kremlin Bicêtre, Assistance Publique Hôpitaux de Paris, Le Kremlin Bicêtre, France; 5 Service de Réanimation Médicale et Médecine Hyperbare, CHU Angers, Angers, France; 6 Sorbonne Université, GRC 30, RESPIRE, UMRS_1166-ICAN, Institute of Cardiometabolism and Nutrition, Service de Médecine Intensive-Réanimation, Assistance Publique-Hôpitaux de Paris (APHP) Hôpital Pitié-Salpêtrière, Paris, France; 7 Service de Médecine Intensive-Réanimation, CHRU Nancy, Nancy, France; 8 Service de Réanimation Polyvalente, Hôpital Privé de l’Ouest Parisien, Ramsay-Générale de Santé, Trappes, France; 9 CESP, INSERM U1018, Equipe Epidémiologie Clinique, Villejuif, France

## Abstract

**Introduction:**

Studies regarding coronavirus disease 2019 (COVID-19) were mainly performed in the initial wave, but some small-scale data points to prognostic differences for patients in successive waves. We therefore aimed to study the impact of time on prognosis of ICU-admitted COVID-19 patients.

**Method:**

We performed a national retrospective cohort study, including all adult patients hospitalized in French ICUs from March 1, 2020 to June 30, 2021, and identified three surge periods. Primary and secondary outcomes were in-hospital mortality and need for invasive mechanical ventilation, respectively.

**Results:**

105,979 critically ill ICU-admitted COVID-19 patients were allocated to the relevant three surge periods. In-hospital mortality for surges 1, 2, and 3 was, respectively, 24%, 27%, and 24%. Invasive mechanical ventilation was the highest level of respiratory support for 42%, 32%, and 31% (*p* < 0.001) over the whole period, with a decline in the use of vasopressors over time. Adjusted for age, sex, comorbidities, and modified Simplified Acute Physiology Score II at ICU admission, time period was associated with less invasive mechanical ventilation and a high risk of in-hospital death. Vaccination against COVID-19 was associated with a lower likelihood of invasive mechanical ventilation (adjusted sub-hazard ratio [aSHR] = 0.64 [0.53–0.76]) and intra-hospital death (aSHR = 0.80, [0.68–0.95]).

**Conclusion:**

In this large database of ICU patients admitted for COVID-19, we observed a decline in invasive mechanical ventilation, vasopressors, and RRT use over time but a high risk of in-hospital death. Vaccination was identified as protective against the risk of invasive mechanical ventilation and in-hospital death.

**Supplementary Information:**

The online version contains supplementary material available at 10.1186/s13613-022-01097-3.

## Introduction

In December 2019, a widespread outbreak of acute respiratory illness—which was due to SARS-CoV-2, a novel coronavirus—emerged from China [1, 2]. The disease, named coronavirus disease 2019 (COVID-19), rapidly spread to over 200 countries worldwide and was officially declared a pandemic by the World Health Organization (WHO) in March 2020 [3]. As of February 2022, more than 130,000 deaths related to COVID-19 have been reported in France [4]. The outbreak caused an unprecedented number of severe cases and intense demand for hospital admissions, especially in intensive care units (ICUs) [3, 5].

The overall pattern of the disease so far has been a series of COVID-19 surges [6]. Although several studies have described the characteristics and the management of COVID-19 patients admitted to an ICU during the first surge of the pandemic [7–9], information regarding the evolution of patients’ characteristics and management over time (i.e., during subsequent surges) is still limited [6, 10]. A decline in the rate of invasive mechanical ventilation use, as well as a rise in in-hospital mortality, was reported in USA between March 2020 and January 2021 [6]. Further studies are still needed to describe trends in ICU care and mortality related to COVID-19.

Using the large French administrative health care database, we aimed to describe the characteristics and the outcomes over time of COVID-19 related critically ill patients. Our primary goal was to compare the characteristics, management, and outcome of patients in French ICUs between the different surges of the pandemic. In addition, we evaluated whether a surge was associated with invasive ventilation and mortality in this population. Finally, we sought to evaluate to what extent vaccination against SARS-CoV-2 could be linked to a decrease in invasive mechanical ventilation and mortality rate.

## Patients and methods

### Study design and participants

This retrospective cohort study used information from the French administrative health care database (Système National des Données de Santé [SNDS]). The SNDS contains data on outpatient care (medical consultation, paramedical interventions, and reimbursed drug dispensation) as well as data from the French hospital discharge database [programme de médicalisation des systems d’informations (PMSI)] collected during hospital stay (admission date, duration, ICD-10 codes for main and associated diagnosis, and medical interventions) [11]. All these data are linked through a unique personal identification number.

We included all adult patients (≥ 18 years old) hospitalized in French ICUs from March 1, 2020 to June 30, 2021, for whom a complete hospital course was available and who had at least one ICD-10 diagnosis code for COVID-19. The complete list of ICD-10 diagnosis codes used to identify patients is provided in Additional file 2: Appendix S1.

Patients with COVID-19 were classified into three groups according to the surges during which they were admitted to the ICU (Additional file 1: Fig. S1) [12]. The first surge occurred between March 1 and June 30, 2020, the second between July 1 and December 31, 2020, and the third from January 1 to June 30, 2021.

### Variables

Age, sex, and Simplified Acute Physiology Score (SAPS) II score [13] at admission were collected for each in-patient stay. We recomposed the Charlson Comorbidity Index [14] based on all ICD-10 diagnoses collected. A number of comorbidities were also collected, namely, arterial hypertension, diabetes mellitus, heart disease, chronic lung disease, cirrhosis, cancer, hematological malignancies, chronic kidney disease, and immunocompromised status. Immunocompromised patients were defined as patients with agranulocytosis, medullar aplasia, immunodeficiency, cancer treated by chemotherapy, or solid organ transplants (ICD-10 diagnosis codes are available in Additional file 2: Appendix S1).

We identified oxygenation and ventilation procedures recorded during hospitalization (according to the French Common Classification of Medical Procedures [CCAM] [15]): invasive mechanical ventilation, non-invasive mechanical ventilation, and high-flow nasal cannula (HFNC) therapy. Patients were classified according to their maximal level of ventilation which was invasive mechanical ventilation followed by non-invasive mechanical ventilation and HFNC therapy. We also identified patients who required prone positioning as well as those who were placed on extracorporeal membrane oxygenation. The list of CCAM codes is available in Additional file 2: Appendix S2. For those requiring invasive mechanical ventilation, time between ICU admission and tracheal intubation was collected.

We used ICD-10 diagnoses to identify complications that occurred during the hospital stay, such as shock-requiring vasopressors, renal replacement therapy (RRT), venous thrombosis events including pulmonary embolism, acute liver failure, and disseminated intravascular coagulation.

Patient outcomes included mechanical ventilation duration, ICU, hospital length of stay (LOS), and vital status at hospital discharge.

For patients admitted after January 1, 2021, we collected vaccination status. A full vaccination scheme was defined as more than 28 days after a single dose of Ad26.COV2-S vaccine (COVID-19 Vaccine Janssen^®^) or more than 7 days after the second dose of vaccine other than Ad26.COV2-S vaccine. A partial vaccination was defined as fewer than 28 days after a single dose of Ad26.COV2-S vaccine or fewer than 7 days after the second and/or after the first dose of vaccine other than Ad26.COV2-S. Patients were considered non-vaccinated if they did not receive any dose of any vaccine against COVID-19.

### Ethics consideration

The SNDS database was created by French law no. 2016-41 dated 26 January 2016 [16]. The purpose of the database is researched through the reuse of claim data gathered after names and social security numbers have been removed. Condition of use and security applying to the database is defined by French government regulation dated 22 March 2017 [17]. As part of its public statistics missions, the Department for Research, Studies, Assessment, and Statistics (DREES) of the French Ministry of Health has permanent access to the SNDS database. An Internet page informs the public about the reuse of the database and their rights according to the European General Data Protection Regulation no. UE 2016/679 dated 27 April 2016 [18].

### Statistical analysis

Characteristics of patients were described as frequencies and percentages for categorical variables and as medians and interquartile ranges (IQRs) for continuous variables. Chi-square test, analysis of variance univariate regression, and logistic univariate regression were used, as appropriate, to compare characteristics of different outbreaks among COVID-19 patients. In all patients of our study, we found 13% of missing values on SAPS II score. We decided not to impute missing data, because we assume that missing data were not at random, and complete case analysis, considering the large number of patients, did not involve a significant change in the confidence intervals of our estimations.

Risk factors of mechanical ventilation were identified through a competing risk framework (i.e., the Fine-Gray model) with ICU discharge alive or death in the ICU without intubation as competing events [19, 20]. The strength of the association between a specific risk factor and the event of interest in the Fine and Gray model is reflected by the sub-hazard ratio (SHR), which is the ratio of hazards associated with the cumulative incidence function in the presence and absence of the risk factor. We first computed SHR for invasive mechanical ventilation and 95% confidence intervals (CIs) associated with each of the risk factors in univariate analysis. Then, we performed a multivariate analysis to adjust for the following predefined potential confounding factors: age, sex, arterial hypertension, diabetes mellitus, heart disease, chronic lung disease, cirrhosis, cancer, hematological malignancies, chronic kidney disease, immunosuppression, and modified SAPS II score. The modified SAPS II score corresponds to SAPS II score without the points related to age. This modification allow us to include both modified SAPS II score and age in the model. Modified SAPS II score was divided into four categories corresponding to the quartiles. No selection covariate procedure was used, because the high number of events limited the risk of overfitting. Proportional hazard assumption was verified with test based on the scaled Schoenfeld residuals.

In the same way, we assessed the association between surge and in-hospital mortality (considering that being discharged alive is a competing risk). To assess the effect of vaccine status, sensitivity analyses were performed in the subgroups of patients admitted after January 1, 2021.

A *P* value < 0.05 was considered significant. Analyses were computed using the SAS 2017 software (SAS Institute, Cary, NC, USA).

## Results

### Patients’ characteristics

During the study period, 105,979 patients critically ill with COVID-19 were admitted to the ICU of 662 hospitals in France: 25,150 (24%), 32,689 (31%), and 48,140 (45%) during the first, second, and third surges, respectively. The majority (*n* = 67,951, 64%) were men and 30% (*n* = 32,044) were younger than 60 years of age. The median SAPS II score was 32 (24–41) (Table 1). Compared with the first surge, patients from the second surge were older (70 [60–78] vs 66 [56–76] years old, *p* < 0.001). Patients from the second and third surges had fewer comorbidities, resulting in a lower Charlson comorbidity index (62% and 73% vs 58% of Charlson comorbidity index equal to 0, *p* < 0.001).

**Table 1:** Characteristics of the study population according to respective COVID-19 surge in France

	All	First surge	Second surge	Third surge	*p *value
	N = 105,979	N = 25,150	N = 32,689	N = 48,140	
*Patient characteristics*					
Age	67 (57–76)	66 (56–76)	70 (60–78)	66 56–75)	< 0.001
Male sex	67,951 (64.12%)	16,662 (66.25%)	21,304 (65.17%)	29,985 (62.29%)	< 0.001
Charlson comorbidity index					
0	69,886 (65.94%)	14,596 (58.04%)	20,321 (62.16%)	34,969 (72.64%)	< 0.001
1–2	24,887 (23.48%)	7180 (28.55%)	8253 (25.25%)	9454 (19.64%)	
–4	6748 (6.37%)	2039 (8.11%)	2454 (7.51%)	2255 (4.68%)	
5 and more	4458 (4.21%)	1335 (5.31%)	1661 (5.08%)	1462 (3.04%)	
*Comorbidities*					
Arterial hypertension	38,530 (36.36%)	10,615 (42.21%)	12,745 (38.99%)	15,170 (31.51%)	< 0.001
Diabetes mellitus	5151 (4.86%)	1512 (6.01%)	1919 (5.87%)	1720 (3.57%)	< 0.001
Heart disease	12,901 (12.17%)	3713 (14.76%)	4702 (14.38%)	4486 (9.32%)	< 0.001
Lung disease	10,732 (10.13%)	3073 (12.22%)	3589 (10.98%)	4070 (8.45%)	< 0.001
Cirrhosis	939 (0.89%)	265 (1.05%)	325 (0.99%)	349 (0.72%)	< 0.001
Cancer	2430 (2.29%)	662 (2.63%)	891 (2.73%)	877 (1.82%)	< 0.001
Hematological malignancies	1893 (1.79%)	483 (1.92%)	759 (2.32%)	651 (1.35%)	< 0.001
Chronic kidney disease	8756 (8.26%)	2618 (10.41%)	3312 (10.13%)	2826 (5.87%)	< 0.001
Immunocompromised status	5018 (4.73%)	1743 (6.93%)	2063 (6.31%)	1212 (2.52%)	< 0.001
SAPS II score	32 (24–41)	32 (24–43)	33 (26–42)	32 (24–40)	< 0.001
*Life support interventions*					
Maximal level of respiratory support					
Invasive mechanical ventilation	36,185 (34.14%)	10,687 (42.49%)	10,358 (31.69%)	15,140 (31.45%)	< 0.001
Non-invasive mechanical ventilation	6749 (6.37%)	766 (3.05%)	2190 (6.70%)	3793 (7.88%)	
High flow nasal canula therapy	19,024 (17.95%)	1743 (6.93%)	6188 (18.93%)	11,093 (23.04%)	
Other oxygenotherapy	44,021 (41.54%)	11,954 (47.53%)	13,953 (42.68%)	18,114 (37.63%)	
Median time between admission and tracheal intubation, in days (Q1–Q3)	2 (0–5)	1 (0–3)	3 (1–6)	2 (1–5)	< 0.001
Tracheostomy	2297 (2.17%)	896 (3.56%)	632 (1.93%)	769 (1.6%)	< 0.001
Prone position	20,231 (19.09%)	5808 (23.09%)	5583 (17.08%)	8840 (18.36%)	< 0.001
*In patients with invasive mechanical ventilation only*	20,231 (55.91%)	4879 (54.35%)	5583 (53.90%)	8840 (58.39%)	< 0.001
Extra-corporeal membrane oxygenation	1125 (1.06%)	344 (1.37%)	297 (0.91%)	484 (1.01%)	< 0.001
Vasopressors use	28,943 (27.31%)	9040 (35.94%)	8453 (25.86%)	11,450 (23.78%)	< 0.001
Renal replacement therapy	7358 (6.94%)	2320 (9.22%)	2297 (7.03%)	2741 (5.69%)	< 0.001
*Clinical outcomes*					
Acute liver failure	1806 (1.70%)	530 (2.11%)	563 (1.72%)	713 (1.48%)	< 0.001
Disseminated intravascular coagulation	446 (0.42%)	121 (0.48%)	143 (0.44%)	182 (0.38%)	0.1055
Pulmonary embolism	7981 (7.53%)	2146 (8.53%)	2293 (7.01%)	3542 (7.36%)	< 0.001
Venous thrombosis	3969 (3.75%)	1302 (5.18%)	1100 (3.37%)	1567 (3.26%)	< 0.001
ICU length of stay, days	7 (3–16)	8 (3–18)	7 (3–15)	7 (3–15)	< 0.001
Hospital length of stay, days	14 (8–24)	14 (7–26)	14 (8–26)	13 (8–23)	< 0.001

SAPS, new simplified acute physiology score; ICU, intensive care unit

### ICU management and complications according to surge

Invasive mechanical ventilation, non-invasive mechanical ventilation, and HFNC therapy were the maximal level of respiratory support for 34% (*n* = 36,185), 6% (*n* = 6749), and 18% (*n* = 19,024) of these COVID-19 patients, respectively (Table 1). The median (IQR) duration of invasive mechanical ventilation was 13 [6–26] days for survivors and 14 [6–26] days for non-survivors.

Prone position and extracorporeal membrane oxygenation were used in 20,231 (19%) and 1125 (1%) patients, corresponding to 56% and 3% of all patients treated with invasive mechanical ventilation, respectively.

Vasopressors were administrated to 27% (*n* = 28,943) of patients (Table 1). RRT was used in 7% (*n* = 7358) of patients. Pulmonary embolism was diagnosed in 8% (*n* = 7981) of patients: 9% during the first surge vs 7% during the second and third surges. During the first surge, vasopressor use, as well as acute kidney injury requiring RRT, were more frequent than during the second and third surges (Table 1).

During the first surge, invasive mechanical ventilation was more frequent than during the second and third surges (42% vs 32% vs 31%, respectively, *p* < 0.001). The third surge was marked by a three-times highest use of HFNC therapy compared to the first surge (7%, vs 19% vs 23%, *p* < 0.001) (Table 1). Among patients with invasive mechanical ventilation, prone position was more frequent during the third surge (58% vs 54% during the first and second surges, *p* < 0.001).

Among patients with COVID-19, the median ICU LOS was 7 [3–16] days and the median hospital LOS was 14 [8–24] days. Of those with invasive mechanical ventilation, ICU LOS was 20 [11–36] days for survivors and 17 [8–36] days for those who did not survive. Of those without invasive mechanical ventilation, ICU LOS was 5 [2–9] and 4 [2–9] days for survivors and non-survivors, respectively.

### Risk factors of invasive mechanical ventilation

A Fine-Gray model revealed that second (adjusted SHR (aSHR) = 0.64 [0.62–0.66]) and third (aSHR = 0.62 [0.61–0.64]) surges were associated with lower risk of invasive mechanical ventilation. The other risk factors of invasive mechanical ventilation were male sex, arterial hypertension, and increased modified SAPS II. Increased age, heart disease, cirrhosis, cancer, hematological malignancies, chronic kidney disease, and immunosuppression were associated with lower risk of invasive mechanical ventilation. Conversely, these covariates were strongly associated with the competing event of death without invasive mechanical ventilation. Table 2 shows the results of this analysis.

**Table 2:** Association between surge and risk of invasive mechanical ventilation

Covariates	Invasive MV		Death without invasive MV		Discharge alive without MV	
	aSHR	95%CI	aSHR	95%CI	aSHR	95%CI
Age (+ 10 years)^a^	0.92	0.912	2.75	2.69–2.81	0.84	0.83–0.84
Male sex	1.23	1.20–1.26	1.08	1.03–1.13	0.84	0.83–0.86
Arterial hypertension	1.24	1.21–1.27	0.75	0.72–0.78	0.95	0.93–0.96
Diabetes mellitus	1.03	0.99–1.08	1.30	1.20–1.42	0.92	0.87–0.96
Heart disease	0.96	0.92–0.99	1.11	1.05–1.17	0.96	0.93–0.99
Lung disease	1.01	0.98–1.04	1.16	1.08–1.23	0.94	0.92–0.97
Cirrhosis	0.84	0.76–0.93	2.33	1.92–2.82	0.89	0.80–0.99
Cancer	0.51	0.47–0.56	2.13	1.93–2.36	1.09	1.02–1.16
Hematological malignancies	0.88	0.82–0.94	1.21	1.07–1.36	0.97	0.90–1.05
Chronic kidney disease	0.67	0.65–0.70	1.21	1.13–1.29	1.19	1.15–1.24
Immunosuppression	0.83	0.79–0.87	1.88	1.73–2.05	0.90	0.86–0.95
Modified SAPS II						
≤ 14	Ref		Ref		Ref	
15–20	2.14	2.06–2.23	1.30	1.23–1.38	0.63	0.62–0.64
21–28	3.70	3.56–3.85	1.51	1.42–1.61	0.41	0.40–0.42
≥ 28	6.96	6.71–7.22	1.70	1.60–1.81	0.18	0.17–0.18
Surge						
First	Ref		Ref		Ref	
Second	0.64	0.62–0.66	1.24	1.17–1.32	1.27	1.24–1.30
Third	0.62	0.61–0.64	1.47	1.39–1.55	1.22	1.19–1.25

aSHR, adjusted sub-hazard ratio; CI, confidence interval; MV, mechanical ventilation; SAPS, simplified acute physiology score

^a^The aSHR of age corresponds to SHR for each 10 years in more

### ICU death outcomes

**Fig. 1:**
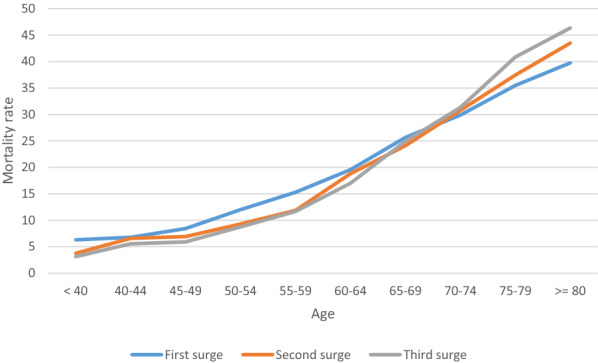
In-hospital mortality by age during each surge of COVID-19. Blue, orange, and gray lines represent age-related mortality rate during the first, second, and third surges, respectively. The mortality rate was higher in people under 70 in the first wave than in the second and third waves. Conversely, the mortality rate increased from wave to wave in patients aged 70 and over

Overall, in-hospital mortality of COVID-19 patients was 25% (*n* = 26,407): 24% (*n* = 6055), 27% (*n* = 8698), and 24% (*n* = 11,654) during the first, second, and third surges, respectively. Among subgroups of patients who required invasive mechanical ventilation, in-hospital mortality was 40% (*n* = 14,516): 35% (*n* = 3789), 44% (*n* = 4576), and 41% (*n* = 6151) during the first, second, and third surges, respectively. The age-specific mortality rate for each COVID-19 surge is shown in Fig. 1. For patients younger than 70, mortality rates were higher during the first surge than in the second and third surges, whereas for those older than 70, the mortality rate during the third surge was higher.

**Fig. 2:**
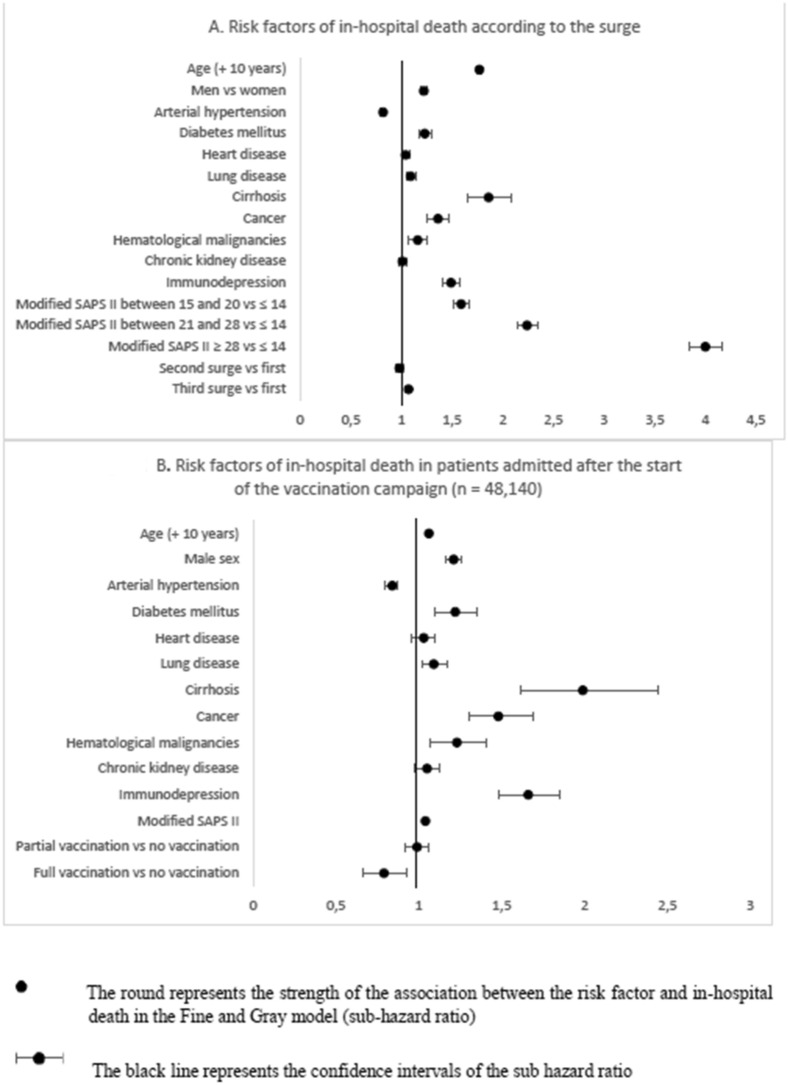
Risk factors of in-hospital death

Competing risk survival analysis revealed that the third surge was associated with a higher risk of in-hospital mortality (Fig. 2A). Other independent risk factors were increasing age, male sex, diabetes mellitus, chronic lung disease, cirrhosis, cancer, hematological malignancies, immunocompromised status, and increasing modified SAPS II score. Conversely, arterial hypertension was associated with a lower risk of in-hospital mortality (Fig. 2A, Additional file 1: Table S1).

### Association between vaccination status and mechanical ventilation and outcome

Of the 48,140 patients of the third surge, 670 (1%) and 4301 (8%) had received a complete or partial vaccination. Characteristics of patients of the third surge, according to their vaccination status, are reported in Additional file 1: Table S2. Invasive mechanical ventilation was less frequent in patients who had completed vaccination programs (19%, *n* = 124) compared to those who had been partially (29%, *n* = 1041) or not vaccinated (32%, *n* = 13,975 (*p* < 0.001). Among patients treated with invasive mechanical ventilation, the duration of mechanical ventilation was shorter in patients with complete vaccination programs (9 [3–14] days) than in those who had only been partially vaccinated (12 [5–23] days) or who were not vaccinated (13 [6–25] days) (*p* < 0.001).

Notably, the Fine–Gray model revealed that full vaccination against SARS-CoV-2 was associated with a lower risk of invasive mechanical ventilation (aSHR = 0.64 [0.53–0.76]), lower risk of in-hospital death (aSHR = 0.80 [0.68–0.95]), and higher risk of discharge alive from the ICU without invasive ventilation (aSHR = 1.32 [1.17–1.49]) (Fig. 2B, Additional file 1: Table S3). Even though incomplete vaccination was not associated with in-hospital death, we observed a significant association with the use of invasive mechanical ventilation (aSHR = 0.93 [0.87–0.99]) and being discharged alive without ventilation (aSHR = 1.08 [1.03–1.14]) (Additional file 1: Table S4).

## Discussion

Of the 105,979 COVID-19 patients admitted to an ICU in France between March 2020 and June 2021, we found a reduction in invasive mechanical ventilation, vasopressors, and RRT use over time. However, after adjustment for confounders such as age, sex, comorbidities, and modified SAPS II, the likelihood of in-hospital death was higher during the third surge compared with the first one. Finally, we confirmed that vaccination was associated with a lower risk of invasive mechanical ventilation treatment and in-hospital death.

During the first surge, invasive mechanical ventilation was more frequent (42%) than during the second (32%) and third surges (31%), where HFNC therapy was more than three times more frequent. This decline in invasive mechanical ventilation use was consistent with findings from a previous study [6]. These differences might be explained by increased experience of COVID-19 management and the evolution of the characteristics of patients admitted to the ICU (especially, an older age during the second surge). In addition, at the beginning of the pandemic, the use of invasive mechanical ventilation was quickly adopted, because non-invasive ventilation or HFNC therapy was considered to put caregivers at a heightened risk of aerosolization [21, 22]. These concerns were assuaged by recent findings showing the clinical benefits of HFNC, as well as its relative safety for caregivers [23, 24]. However, the usefulness of non-invasive ventilation is still a matter of debate [24]. Thus, between the first and the second surge in France, French recommendations regarding the management of COVID-19 patients were modified by advocating corticosteroids and non-invasive ventilation support [25].

Using the first surge as a reference and after adjusting for age, comorbidities, and severity at ICU admission, the Fine and Gray model confirmed that the third surge was still strongly associated with a poorer outcome. In France, during the third surge, alpha (lineage B.1.1.7) was the predominant variant of concern. This has been associated with higher mortality in other countries [26, 27]. The other hypothesis possibly incriminated in the poorer outcome of the third surge related to the excess of strain in ICUs [28]. Although no statistic was available concerning the excess of work observed in ICUs, the peak of patients hospitalized in ICU in France were 17.893, 17.800, and 18.474, respectively, during surge 1, 2, and 3. If these peaks were not different according the first two surges, the higher number of patients admitted in ICU during the third surge may have been accompanied by a higher strain in these units. Consistent with the literature [29–33], we identified that age, male sex, comorbidities, and high SAPS II score at ICU admission are risk factors for poor outcomes. However, besides vital status signs, competing risk analysis highlighted that older age and several comorbidities, such as cirrhosis, cancer, chronic kidney disease, and immunosuppression, were associated with a lower risk of mechanical ventilation. However, caution is required so as not to misinterpret these results, since they are directly associated with the decision to withhold life-sustaining therapy in the frailest patients, as illustrated by the strong association of these same comorbidities with death without mechanical ventilation.

Because an active vaccination campaign began in France in December 2020, we could observe the impact of vaccination on outcomes during the third surge. Compared to non-vaccinated patients, vaccination against SARS-Cov-2 was associated with a lower likelihood of invasive mechanical ventilation, in-hospital death, and a higher risk of being discharged alive without ventilation. These results are consistent with another study [34] including 1197 patients hospitalized for COVID-19, which reported that death or invasive mechanical ventilation by day 28 was associated with a decreased likelihood of vaccination (12.0% vs 24.7%; aOR, 0.33; 95% CI 0.19–0.58). Our results reinforce the positive impact of vaccination on severity, even when vaccinated patients have been infected.

To the best of our knowledge, this study is the largest focus on critically ill patients in this context. Moreover, we were able to analyze patients admitted after the beginning of the vaccine campaign which allowed us to assess the effect of vaccination on ICU outcomes. However, our study has some limitations. First, due to the retrospective design, we could not confirm the causality of the associations we observed. However, the high number of patients included in our study limit the bias of overfitting. Second, biological information during the ICU stay is not collected in the SNDS, in which case, arterial blood gas could have been used as a surrogate for respiratory failure severity, whereas SAPS II expressed overall severity, including the respiratory component. Third, information concerning the treatment used in the ICU (such as corticosteroids, anticoagulants, and antivirals or the withholding of life-sustaining therapy) was not collected in the SNDS. Indeed, between the first surge and the others, expert recommendations were published advocating the use of corticosteroids and anticoagulants in critically ill patients with COVID-19 [25]. Unfortunately, the information was not available in our study and we therefore cannot assess the impact of these treatments on the prognosis of patients. Fourth, information about the variant of concern identified for each COVID case was not routinely recorded at the time of the pandemic. Finally, if the peak of ICU hospitalization was available, these data are too limited to be approach as a reliable proxy of excess of strain in ICU for two major reasons. First, COVID-19 pandemic has particularly involved medical and paramedical staff, accompanied by an excess of professional burnout and a possible decrease in the medical and nursing professional workforce [35]. Unfortunately, statistics describing available caregivers over time are not available. Second, the peak of ICU hospitalization is a national level information, and the intensity of the COVID-19 pandemic was not homogeneous in the whole territory.

## Conclusion

From a population-based study involving 105,979 COVID-19 patients admitted to an ICU between March 2020 and July 2021, we reported a decline in invasive mechanical ventilation, vasopressors, and RRT use over time. We also found a rise in the risk of in-hospital mortality during the third compared with the first surge. Finally, vaccination was associated with a lower risk of invasive mechanical ventilation and in-hospital death.

## Supplementary Information

**Additional file 1: Table S1.** Association between surge and in-hospital death. **Table S2.** Characteristics of patients of the third surge according to their vaccination status. **Table S3.** Association between vaccination status and the use of invasive mechanical ventilation. **Table S4.** Association between vaccination and in-hospital mortality. **Figure S1.** Admission of patients with COVID-19 in ICU between March 2020 and July 2021. **Figure S2.** Cumulative incidence curve of invasive mechanical ventilation (A) and discharge alive without invasive mechanical ventilation (B) according to vaccine status.

**Additional file 2: Appendix S1.** List of ICD-10 diagnosis codes used. **Appendix S2.** List of CCAM codes used.

## Data Availability

The data that support the findings of this study are available from the Department for Research, Studies, Assessment, and Statistics (DREES) of the French Ministry of Health, but restrictions apply to the availability of these data and so are not publicly available.
