# Supplementary material for: Trends in clinical characteristics and outcomes of all critically ill COVID-19 adult patients hospitalized in France between March 2020 and June 2021: a national database study
Source: Ann Intensive Care. 2023 Jan 12;13:2. doi: 10.1186/s13613-022-01097-3 (PMC9834443; doi:10.1186/s13613-022-01097-3)

**Table S1.** Association between surge and in-hospital death

|  | **In-hospital death** | | | |
| --- | --- | --- | --- | --- |
|  | Non-aSHR | 95%CI | aSHR | 95%CI |
| Age (+10 years)* | 1.63 | 1.62 - 1.65 | 1.77 | 1.75-1.79 |
| Gender |  |  |  |  |
| Female | Ref |  | Ref |  |
| Male | 1.16 | 1.13 - 1.19 | 1.22 | 1.19-1.25 |
| Arterial hypertension | 1.12 | 1.09 - 1.15 | 0.82 | 0.79-0.84 |
| Diabetes mellitus | 1.48 | 1.41 - 1.55 | 1.23 | 1.17-1.30 |
| Heart disease | 1.51 | 1.46 - 1.56 | 1.04 | 1.00-1.08 |
| Lung disease | 1.16 | 1.12 - 1.20 | 1.09 | 1.05-1.14 |
| Cirrhosis | 1.69 | 1.52 - 1.87 | 1.86 | 1.65-2.09 |
| Cancer | 1.99 | 1.87 - 2.12 | 1.36 | 1.26-1.47 |
| Hematological malignancies | 1.95 | 1.82 - 2.09 | 1.16 | 1.07-1.25 |
| Chronic kidney disease | 1.66 | 1.59 - 1.72 | 1.01 | 0.97-1.06 |
| Immunodepression | 1.80 | 1.72 - 1.89 | 1.49 | 1.41-1.58 |
| Modified SAPS II |  |  |  |  |
| ≤ 14 | Ref |  | Ref |  |
| 15-20 | 1.54 | 1.47-1.61 | 1.59 | 1.52-1.67 |
| 21-28 | 2.20 | 2.11_2.30 | 2.24 | 2.15-2.35 |
| ≥ 28 | 3.87 | 3.72-4.03 | 4.00 | 3.84-4.17 |
| Surge |  |  |  |  |
| First | Ref |  | Ref |  |
| Second | 1.11 | 1.07 - 1.15 | 0.98 | 0.95-1.02 |
| Third | 0.99 | 0.96 - 1.03 | 1.07 | 1.03-1.10 |
| aSHR, adjusted sub-hazard ratio; CI, confidence interval; SAPS, Simplified Acute Physiology Score  * The aSHR of age correspond to SHR for each 10 years in more | | | | |

**Table S2**. Characteristics of patients of the third surge according to their vaccination status

|  | **Full vaccination** | **Partial vaccination** | **No vaccination** | ***p*** |
| --- | --- | --- | --- | --- |
|  | N=669 | N=3638 | N=43 833 |  |
| **Patient characteristics** |  |  |  |  |
| **Age, years** | 75.09 (± 12.88) | 70.96 (± 11.50) | 64.09 (±14.68) | *<.0001* |
| **Male gender** | 392 (58.59 %) | 1275 (35.05 %) | 27230 (62.12 %) | *0.0005* |
| **Charlson comorbidity index** |  |  |  |  |
| 0 | 446 (66.67 %) | 2530 (69.54 %) | 31992 (72.99 %) | *<.0001* |
| 1-2 | 143 (21.38 %) | 790 (21.72 %) | 8523 (19.44 %) |  |
| 3-4 | 36 (5.38 %) | 177 (4.87 %) | 2059 (4.70 %) |  |
| 5 and more | 44 (6.58 %) | 141 (3.88 %) | 1259 (2.87 %) |  |
| **Comorbidities** |  |  |  |  |
| Arterial hypertension | 189 (28.25 %) | 1224 (33.64 %) | 13757 (31.39 %) | *0.0035* |
| Diabetes mellitus | 25 (3.74 %) | 163 (4.48 %) | 1532 (3.50 %) | *0.0086* |
| Heart disease | 72 (10.76 %) | 370 (10.17 %) | 4044 (9.23 %) | *0.0736* |
| Lung disease | 36 (5.38 %) | 349 (9.59 %) | 3685 (8.41 %) | *0.0008* |
| Cirrhosis | 2 (0.30 %) | 32 (0.88 %) | 315 (0.72 %) | *0.2322* |
| Cancer | 31 (4.63 %) | 80 (2.20 %) | 766 (1.75 %) | *<.0001* |
| Hematological malignancies | 40 (5.98 %) | 80 (2.20 %) | 531 (1.21 %) | *<.0001* |
| Chronic kidney disease | 99 (14.80 %) | 305 (8.38 %) | 2422 (5.53 %) | *<.0001* |
| Immunodepression | 73 (10.91 %) | 126 (3.46 %) | 1013 (2.31 %) | *<.0001* |
| **SAPS II score** | 32 (15-41) | 31 (22-40) | 29 (20-38) | *0.0001* |
| **Life support interventions** |  |  |  |  |
| **Maximal level of respiratory support** |  |  |  |  |
| Invasive mechanical ventilation | 124 (18.54 %) | 1041 (28.61 %) | 13975 (31.88 %) | *<.0001* |
| Non-invasive mechanical ventilation | 46 (6.88 %) | 268 (7.37 %) | 3479 (7.94 %) |  |
| High flow nasal canula therapy | 83 (12.41 %) | 801 (22.02 %) | 10209 (23.29 %) |  |
| Other oxygenotherapy | 416 (62.18 %) | 1528 (42.00 %) | 16170 (36.89 %) |  |
| **Tracheotomy** | 3 (0.45 %) | 40 (1.10 %) | 726 (1.66 %) | *0.0032* |
| **Prone position** | 57 (8.52 %) | 603 (16.58 %) | 8180 (18.66 %) | *<.0001* |
| In patients with invasive mechanical ventilation only | 57 (45.97 %) | 438 (57.93 %) | 5795 (58.53 %) | *0.0175* |
| **Extra-corporeal membrane oxygenation** | 1 (0.15 %) | 19 (0.52 %) | 464 (1.06 %) | *0,0015* |
| **Vasopressors use** | 102 (15.25 %) | 814 (22.37 %) | 10534 (24.03 %) | *<.0001* |
| **Renal replacement therapy** | 46 (6.88 %) | 233 (6.40 %) | 2462 (5.62 %) | *0.0593* |
| **Clinical outcomes** |  |  |  |  |
| **Acute liver failure** | 9 (1.35 %) | 53 (1.46 %) | 651 (1.49 %) | *0.9492* |
| **Disseminated intravascular coagulation** | 3 (0.45 %) | 12 (0.33 %) | 167 (0.38 %) | *0.8514* |
| **Pulmonary embolism** | 25 (3.74 %) | 222 (6.10 %) | 3295 (7.52 %) | *<.0001* |
| **Venous thrombosis** | 11 (1.64 %) | 103 (2.83 %) | 1453 (3.31 %) | *0.0176* |
| **ICU length of stay, days** | 5 (2-11) | 7 (3-14) | 7 (4-15) | *<.0001* |
| **Hospital length of stay, days** | 11 (6-19) | 13 (7-21) | 13 (8-23) | *<.0001* |

**Table S3.** Association between vaccination status and the use of invasive mechanical ventilation

|  | **Invasive MV** | | **Death without invasive MV** | | **Discharge alive without MV** | |
| --- | --- | --- | --- | --- | --- | --- |
| **Covariates** | aSHR | 95%CI | aSHR | 95%CI | aSHR | 95%CI |
| Age (+10 years)* | 0.94 | 0.93-0.95 | 2.84 | 2.76-2.93 | 0.82 | 0.81-0.83 |
| Gender |  |  |  |  |  |  |
| Female | Ref |  | Ref |  | ref |  |
| Male | 1.16 | 1.12-1.20 | 1.12 | 1.05-1.19 | 0.89 | 0.86-0.91 |
| Arterial hypertension | 1.23 | 1.18-1.27 | 0.77 | 0.72-0.82 | 0.97 | 0.94-1.00 |
| Diabetes mellitus | 1.06 | 0.98-1.15 | 1.36 | 1.18-1.57 | 0.88 | 0.81-0.95 |
| Heart disease | 1.03 | 0.97-1.09 | 1.05 | 0.96-1.15 | 0.90 | 0.85-0.95 |
| Lung disease | 1.09 | 1.03-1.15 | 1.08 | 0.97-1.20 | 0.90 | 0.86-0.94 |
| Cirrhosis | 0.96 | 0.81-1.13 | 2.56 | 1.86-3.54 | 0.75 | 0.63-0.90 |
| Cancer | 0.56 | 0.49-0.65 | 2.19 | 1.85-2.58 | 0.98 | 0.88-1.09 |
| Hematological malignancies | 0.94 | 0.83-1.06 | 1.27 | 1.03-1.55 | 0.93 | 0.81-1.06 |
| Chronic kidney disease | 0.75 | 0.69-0.80 | 1.19 | 1.07-1.32 | 1.10 | 1.03-1.18 |
| Immunodepression | 0.87 | 0.78-0.96 | 1.95 | 1.67-2.28 | 0.82 | 0.74-0.91 |
| Modified SAPS II |  |  |  |  |  |  |
| ≤ 14 | Ref |  | Ref |  | Ref |  |
| 15-20 | 1.90 | 1.79-2.02 | 1.24 | 1.14-1.36 | 0.67 | 0.65-0.69 |
| 21-28 | 3.22 | 3.04-3.42 | 1.47 | 1.35-1.61 | 0.45 | 0.44-0.47 |
| ≥ 28 | 6.01 | 5.68-6.36 | 1.71 | 1.56-1.87 | 0.22 | 0.21-0.23 |
| Vaccination status |  |  |  |  |  |  |
| Not vaccinated | Ref |  | Ref |  | Ref |  |
| Partial vaccination | 0.93 | 0.87-0.99 | 1.00 | 0.90-1.10 | 1.08 | 1.03-1.14 |
| Full vaccination | 0.64 | 0.53-0.76 | 0.86 | 0.70-1.06 | 1.32 | 1.17-1.49 |
| aSHR. adjusted sub-hazard ratio; CI. confidence interval; MV. mechanical ventilation; SAPS. Simplified Acute Physiology Score  * The aSHR of age correspond to SHR for each 10 years in more | | | | | | |

**Table S4.** Association between vaccination and in-hospital mortality

|  | **In-hospital death** | | | |
| --- | --- | --- | --- | --- |
|  | Non-aSHR | 95%CI | aSHR | 95%CI |
| Age (+10 years)* | 1.74 | 1.71 - 1.76 | 1.86 | 1.83-1.90 |
| Sex |  |  |  |  |
| Female | Ref |  | Ref |  |
| Male | 1.13 | 1.09 - 1.17 | 1.22 | 1.16-1.27 |
| Arterial hypertension | 1.14 | 1.10 - 1.18 | 0.83 | 0.79-0.87 |
| Diabetes mellitus | 1.55 | 1.42 - 1.68 | 1.23 | 1.12-1.36 |
| Heart disease | 1.60 | 1.52 - 1.69 | 1.03 | 0.96-1.10 |
| Lung disease | 1.17 | 1.10 - 1.24 | 1.09 | 1.01-1.16 |
| Cirrhosis | 1.81 | 1.53 - 2.14 | 2.15 | 1.76-2.64 |
| Cancer | 2.25 | 2.03 - 2.50 | 1.40 | 1.23-1.61 |
| Hematological malignancies | 2.16 | 1.93 - 2.42 | 1.25 | 1.09-1.43 |
| Chronic kidney disease | 1.80 | 1.69 - 1.92 | 1.06 | 0.98-1.15 |
| Immunodepression | 2.27 | 2.08 - 2.46 | 1.65 | 1.48-1.85 |
| Modified SAPS II |  |  |  |  |
| ≤ 14 | Ref |  | Ref |  |
| 15-20 | 1.39 | 1.30-1.48 | 1.48 | 1.39-1.59 |
| 21-28 | 2.00 | 1.87-2.13 | 2.08 | 1.95-2.22 |
| ≥ 28 | 3.58 | 3.37-3.80 | 3.69 | 3.47-3.93 |
| Vaccination status |  |  |  |  |
| Not vaccinated | Ref |  | Ref |  |
| Partial vaccination | 1.32 | 1.24 - 1.41 | 1.01 | 0.94-1.08 |
| Full vaccination | 1.40 | 1.22 - 1.62 | 0.80 | 0.68-0.95 |
| aSHR. adjusted sub-hazard ratio; CI. confidence interval; SAPS. Simplified Acute Physiology Score  * The aSHR of age correspond to SHR for each 10 years in more | | | | |

**Figure S1.** Admission of patients with Covid-19 in ICU between March 2020 and July 2021

Third surge

Second surge

First surge

**Figure S2.** Cumulative incidence curve of invasive mechanical ventilation (A) and discharge alive without invasive mechanical ventilation (B) according to vaccine status


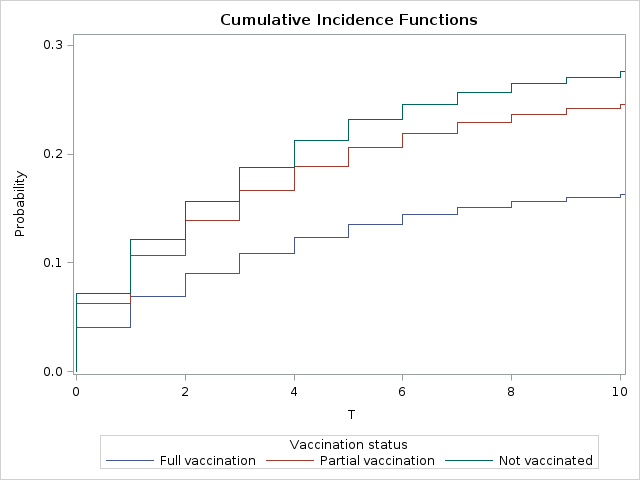


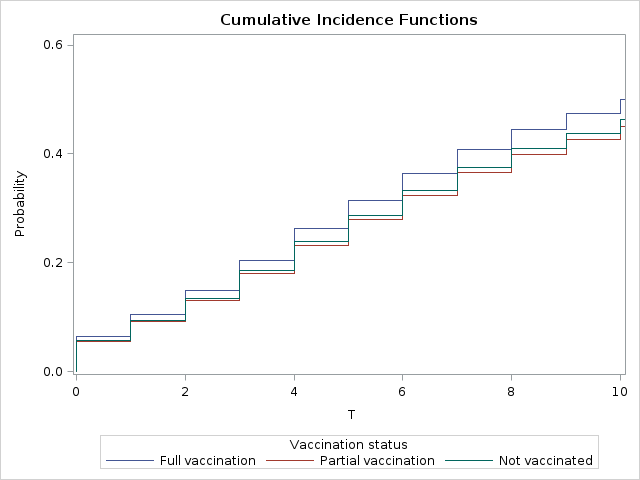

Supplement: Supplementary file 1 — Additional file 1: Table S1. Association between surge and in-hospital death. Table S2. Characteristics of patients of the third surge according to their vaccination status. Table S3. Association between vaccination status and the use of invasive mechanical ventilation. Table S4. Association between vaccination and in-hospital mortality. Figure S1. Admission of patients with COVID-19 in ICU between March 2020 and July 2021. Figure S2. Cumulative incidence curve of invasive mechanical ventilation (A) and discharge alive without invasive mechanical ventilation (B) according to vaccine status. [file 13613_2022_1097_MOESM1_ESM.docx]
