# Supplementary material for: Trends in clinical characteristics and outcomes of all critically ill COVID-19 adult patients hospitalized in France between March 2020 and June 2021: a national database study
Source: Ann Intensive Care. 2023 Jan 12;13:2. doi: 10.1186/s13613-022-01097-3 (PMC9834443; doi:10.1186/s13613-022-01097-3)
Supplement: Supplementary file 2 — Additional file 2: Appendix S1. List of ICD-10 diagnosis codes used. Appendix S2. List of CCAM codes used. [file 13613_2022_1097_MOESM2_ESM.docx]

**Additional file 2**

**Appendix S1. List of ICD-10 diagnosis codes used**

# ICD-10 codes for COVID-19

U0710 - COVID-19, respiratory presentation, identified virus

U0711 - COVID-19, respiratory presentation, virus not identified

U0714 - COVID-19, other clinical presentations, identified virus

U0715 - COVID-19, other clinical presentations, virus not identified

ICD-10 CODES FOR IMMUNOSUPPRESION

B20, B21, B22, B23, B24 – HIV infection

D61 - medullar aplasia

D70 - agranulocytosis

D80, D81, D82, D83, D84 – immunodeficiency

Z94 - solid organ transplants

Z511 - cancer treated by chemotherapy

# ICD-10 codes for COMPLICATIONS

J80 – Adult respiratory distress syndrome

N17.x – Acute renal failure

K72.0 – Acute and subacute hepatic failure

D65 – Disseminated intravascular coagulation (defibrination syndrom)

I80.x – Phlebitis and thrombophlebitis

I26.x – Pulmonary embolism

**Appendix S2. List of CCAM codes used**

Invasive mechanical ventilation

GLLD004 - Intratracheal mechanical ventilation with positive end expiratory pressure [PEP] greater than 6 and/or FiO2 greater than 60%, with alternating prone position technique per 24 hours

GLLD007 - Mechanical ventilation with separate lungs, per 24 hours

GLLD008 - Intratracheal mechanical ventilation with positive end expiratory pressure [PEP] greater than 6 and/or FiO2 greater than 60%, per 24 hours

GLLD009 - High Frequency Oscillating Ventilation, Per 24 Hours

GLLD015 - Intratracheal Mechanical Ventilation with Positive Expiratory Pressure [PEP] less than or equal to 6 and FiO2 less than or equal to 60%, per 24 hours

Non-invasive mechanical ventilation

GLLD012 - Continuous mechanical ventilation with face mask, per 24 hours

High*-*flow nasal cannula (HFNC) therapy

GLLD003 - Spontaneous ventilation by face mask, nasal cannula or nasopharyngeal tube, without pressure support, with positive expiratory pressure [CV-PEP] [Continuous positive airway pressure] [CPAP], per 24 hours

Tracheotomy

GLLD013 - Spontaneous ventilation on tracheostomy during weaning from mechanical ventilation, per 24 hours

Prone position

GLLD004 - Intratracheal mechanical ventilation with positive end expiratory pressure [PEEP] greater than 6 and/or FiO2 greater than 60%, with alternating prone position technique per 24 hours

Extracorporeal membrane oxygenation (ECMO)

GLJF010 - Extracorporeal carbon dioxide [CO2] purification, per 24 hours

Catecholamines use

EQLF001 - Continuous intravenous injection of dobutamine or dopamine at a rate of less than 8 micrograms per kilogram per minute [µg/kg/min], or dopexamine outside the neonatal period, per 24 hours

EQLF003 - Continuous intravenous injection of dobutamine or dopamine at a rate greater than 8 micrograms per kilogram per minute [µg/kg/min], adrenaline or noradrenaline outside the neonatal period, per 24 hours

Renal replacement therapy

JVJB002 - Extrarenal purification by peritoneal dialysis for acute renal failure, per 24 hours

JVJF002 - Extrarenal purification by hemodialysis, hemodiafiltration or discontinuous hemofiltration for acute renal failure, per 24 hours

JVJF003 - Extrarenal purification session by hemoperfusion

JVJF005 - Extrarenal purification by hemodialysis, hemodiafiltration or continuous hemofiltration for acute renal failure, per 24 hours
